# Effect of virtual reality distraction on anxiety, pain and discomfort during unsedated gastroscopy: Randomized controlled trial

**DOI:** 10.1055/a-2794-0424

**Published:** 2026-02-17

**Authors:** Zita Bouman, Froukje De Vries, Mattanja De Ruiter, Anneloes Bakker, Adriaan CITL Tan

**Affiliations:** 1 6030 Medical Psychology, Canisius Wilhelmina Hospital, Nijmegen, The Netherlands; 2 561033 Psychiatry, Pro Persona GGz Adults Nijmegen, Nijmegen, The Netherlands; 3 8189 Psychiatry, Vincent Van Gogh Instituut, Venray, The Netherlands; 4 6030 Gastroenterology & Hepatology, Canisius Wilhelmina Hospital, Nijmegen, The Netherlands

**Keywords:** Quality and logistical aspects, Sedation and monitoring, Preparation

## Abstract

**Background and study aims:**

Esophagogastroduodenoscopy is a widely used medical examination. Despite the short duration of this procedure, many patients experience anxiety, pain, and discomfort. The aim of this study was to examine effects of virtual reality (VR) distraction on anxiety, pain, and discomfort during unsedated gastroscopy.

**Patients and methods:**

Thirty-nine patients in the intervention group wore VR glasses 10 minutes before and during the gastroscopy. Fifty patients in the control group received care as usual. Anxiety and pain levels were measured with the STAI-DY and NRS before, during, and after the procedure. Moreover, comfort level was reported by an accompanying nurse during the procedure. The last outcome was self-reported willingness to undergo unsedated vs. sedated gastroscopy in the future.

**Results:**

The VR and control non-VR groups were comparable in terms of age, gender, education level, and self-reported general health, anxiety, and pain levels. No significant differences were observed in levels of anxiety, pain, and discomfort during the endoscopy. There was no difference in medical outcome and willingness to undergo unsedated vs. sedated gastroscopy in the future.

**Conclusions:**

VR distraction did not objectively reduce patient anxiety, pain, or discomfort before, during, or after the procedure. Moreover, willingness to undergo the same procedure without sedation was the same for the VR and control groups. Future research is needed to explore whether selected groups of patients may benefit from VR distraction.

## Introduction

Esophagogastroduodenoscopy (EGD) is a widely used medical examination. Despite the short duration of this procedure, many patients experience anxiety, pain, and discomfort 1 . Because of this, in the Netherlands, an increasing number of patients are opting for (light) sedation. Light sedation is relatively safe, but in rare instances, can have adverse effects. Especially for patients with severe comorbidities, sedation is related to higher risk of choking after which aspiration (pneumonia) can occur. Overdose of sedation can also lead to areflexia, apnea, hypotension, cardiorespiratory depression, or even coma 2 . Moreover, gastroscopy with sedation is expensive and time consuming 3 . It requires a stay in the recovery room and monitoring of patients during and after the procedure. Furthermore, due to safety and regulation issues, patients should be seen beforehand to check for factors that may influence sedation. This increases the cost of gastroscopy under sedation, whereas gastroscopy without sedation is a relatively simple and short procedure. In our hospital, gastroscopy without sedation costs approximately €300, and with sedation is 2.2 times more expensive at approximately €670. Therefore, the main driver for the costs is sedation. Because the costs of healthcare in the Netherlands have increased exponentially 4 , it is important to assess the indication for gastroscopies, but also to reduce the costs of the gastroscopies that are performed. If patients are less anxious and feel more comfortable, more patients may be willing to opt for gastroscopy without sedation, which would substantially reduce costs in healthcare.

Several studies have examined non-pharmacological interventions to decrease anxiety and discomfort before and during gastroscopy, such as preparing patients with a brief intervention 5 , listening to music 6 7 8 , or watching images 9 . Moreover, use of virtual reality (VR) distraction has been shown as a promising, noninvasive, and well-accepted intervention during various endoscopic procedures such as colonoscopy (not gastroscopy) 10 11 12 13 .

To the best of our knowledge, only one study to date has examined VR distraction during gastroscopy. This study demonstrated that VR distraction did not reduce patient pain measures (VAS 0–10 scale), heart rate, or blood pressure during gastroscopy 14 . Although physiological measures (heart rate and blood pressure) were examined, subjective measures are considered the definitive standard for patient evaluation because physiological measures are generally poorly correlated with anxiety 15 . In this study, the authors did not study validated psychological tests. Thus, the primary aim of the present study was to examine the effect of VR distraction on reducing patient self-reported pain and anxiety as measured with psychological questionnaires. Moreover, this study also evaluated comfort as assessed by an endoscopy nurse and self-reported willingness to undergo gastrointestinal endoscopy without sedation in the future.

## Patients and methods

### Trial design

This randomized controlled trial (RCT) was conducted at one general community hospital in the Netherlands, namely the Canisius Wilhelmina Hospital. The study was funded by ZonMw, the Netherlands Organization for Health research and Development and the Science fund of Canisius Wilhelmina Hospital (RvB 21.038/CK/GvE). The research protocol was approved by the Local Review Commission (LTC) of Canisius Wilhelmina Hospital (047–2021). All patients provided informed consent digitally before enrollment.

Patients or the public were not involved in the design, or conduct, or reporting, or dissemination plans of our research.

### Study population

From July 2021 to August 2023, one nurse endoscopist screened and enrolled patients who were referred for unsedated gastrointestinal endoscopy. Patients were eligible for the study if they were 18 years or older and had sufficient knowledge of the Dutch language to complete psychological questionnaires and follow instructions independently. Exclusion criteria were pregnancy, recent gastrointestinal endoscopy (< 5 years), visual and/or auditory impairments, and diagnosis of balance disorders or epilepsy.

Patients who met the above criteria and were willing to participate received more information about the study. After digital submission via an online survey platform, Enalyzer, we randomized patients who underwent unsedated gastroendoscopy to a VR group and control group by using sequentially assigned sealed randomization envelopes that were opened by a colleague not involved in the trial. Blinding of patients was not possible because the VR group had to wear the VR glasses. Patients were informed about which group they were allocated to on the day of the procedure.

### Study interventions

The Pico G2 4K Enterprise was used to generate VR distraction. This model is a VR headset that projects 360-degree video into two independent lenses. The VR headset is positioned on the head with elastic straps that consist of non-porous materials to fit hygienic purposes. In this study we used the application VRelax (version 1.3.41.0), which consists of distracting nature views. Patients were offered a choice of three videos featuring swimming dolphins, cows in the Alps, or sunrise near a lake. This video content was selected based on the relaxing character and because it did not invite patients to move their heads. Besides visual input, the VR content also had natural audio effects during which communication with the medical team still was possible.

### Study design

Five to 6 days before the gastroscopy (T1), patients were invited by email to fill in baseline questionnaires that started with informed consent and are followed by questions concerning demography and validated questionnaires concerning general health (RAND-36), level of pain (numeric rating scale [NRS]) and anxiety (State-Trait Anxiety [STAI]). Data were collected using Enalyzer (2023). If patients did not respond, they received a reminder by phone.

On the day of gastroscopy (T2), patients filled in validated questionnaires on anxiety (NRS and STAI) and pain (NRS) on a tablet approximately 15 minutes before the start of the gastroscopy. Patients in the intervention group also tested the VR glasses before gastroscopy for 10 minutes to get accustomed to them.

During the gastroscopy (T3) two nurses observed patient comfort level (0–10) and time of the procedure.

After gastroscopy (T4), patients completed a set of questionnaires on a tablet including anxiety (NRS and STAI), pain (NRS) and willingness to undergo a next procedure without sedation and/or VR.

Three months after gastroscopy (T5), patients received an email with questions regarding patient satisfaction (NRS) and willingness to undergo a next procedure without sedation and/or VR.

### Measurements

#### Gastroscopy procedures

Gastroscopy was performed by two experienced endoscopists using the Olympus HD gastroscopes. In accordance with routine practice at our hospital, no topical throat anesthesia was administered prior to unsedated gastroscopy. The time of duration of passage was measured in seconds. The upper gastrointestinal tract endoscopy results were categorized into three categories: no abnormality, not a clinically relevant finding, and a clinically relevant finding 16 .

#### Sociodemographic characteristics

Age, gender, and level of education were self-reported by participants. To classify level of education, the Dutch Verhage scale was used 17 . The seven categories were merged into three ordinal categories: low educational level (Verhage 1 until 4), middle educational level (Verhage 5), and high educational level (Verhage 6 and 7).

#### General health

The general health status of patients was measured using the RAND-36 questionnaire 18 .

#### Patient anxiety, pain, comfort and willingness to undergo unsedated gastrointestinal endoscopy

To measure level of anxiety of patients before and after the procedure, two measures were used. Anxiety was measured with the widely used STAI. The STAI consists of two self-evaluation questionnaires. First is the state portion, which measures immediate emotional state caused by concern or tension that may change over time. Second is the trait portion, which measures a personality of feeling fearful in a safe situation. In this study, the STAI trait questionnaire is only used to compare baseline characteristics of the groups and the state questionnaire is used at the different time points. Both questionnaires consist of 20 items and have a total score from 20 for absence of anxiety to 80 for high anxiety 19 . In addition to assessing anxiety using the STAI, patient-reported measures included scales for anxiety, pain, patient comfort, and willingness to undergo unsedated vs sedated gastrointestinal endoscopy in the future. The STAI provided a validated and standardized measure of both state and trait anxiety, whereas the scales offered more immediate, subjective ratings of patient experiences. NRS scores for anxiety range from 0 which represents no anxiety and to 10 being the highest imaginable anxiety 20 . Also, patient pain was measured using an 11-point NRS in which 0 represents no pain and 10 the highest imaginable pain 21 . Moreover, patient comfort was measured by an endoscopy nurse using an 11-point NRS in which 0 is severe discomfort and 10 is comfortable 22 . Patient willingness to undergo unsedated vs sedated gastrointestinal endoscopy in the future was measured using an 11-point NRS in which 0 was unwilling to undergo without sedation and 10 was willing to undergo without sedation. Together, these complementary measures allowed for a comprehensive assessment of both psychological and physical experiences relevant to the study outcomes.

## Statistical analyses

For power analyses, we expected the mean difference between the groups with and without VR to be 30% (80% in the group with VR and 50% in the group without VR). With one-sided α of 0.05, and power of 80%, we estimated that a sample size of 40 would be sufficient. We planned to recruit an additional eight patients per group.

We used the V.27 of IBM SPPS Statistics software for statistical analyses. Due to use of the software on T1 and T2, there were no missing data because patients had to answer all questions to get to the next page. The response rate on T4 and T5 was somewhat lower.

We presented baseline characteristics by using frequencies for categorical variables and means and standard deviations or medians and interquartile ranges for continuous variables.

The primary analyses were performed with repeated measures ANOVA. For pairwise post-hoc comparisons, Bonferroni correction was applied to adjust for multiple testing. For the secondary analyses several measures were applied. Univariate ANOVAs were used for continuous variables with partial eta squared (η²p) reported as effect size measures. Non-parametric comparisons were performed using the Mann-Whitney U test, wit effect size *r* calculated from the standardized test statistic ( *Z* ). Chi square tests were used to analyze categorical variables, with odds ratios (ORs) with 95% confidence intervals (CIs) reported as effect size measures. *P* <.0.5 was considered statistically significant.

## Results

### Patients

**Fig. 1:**
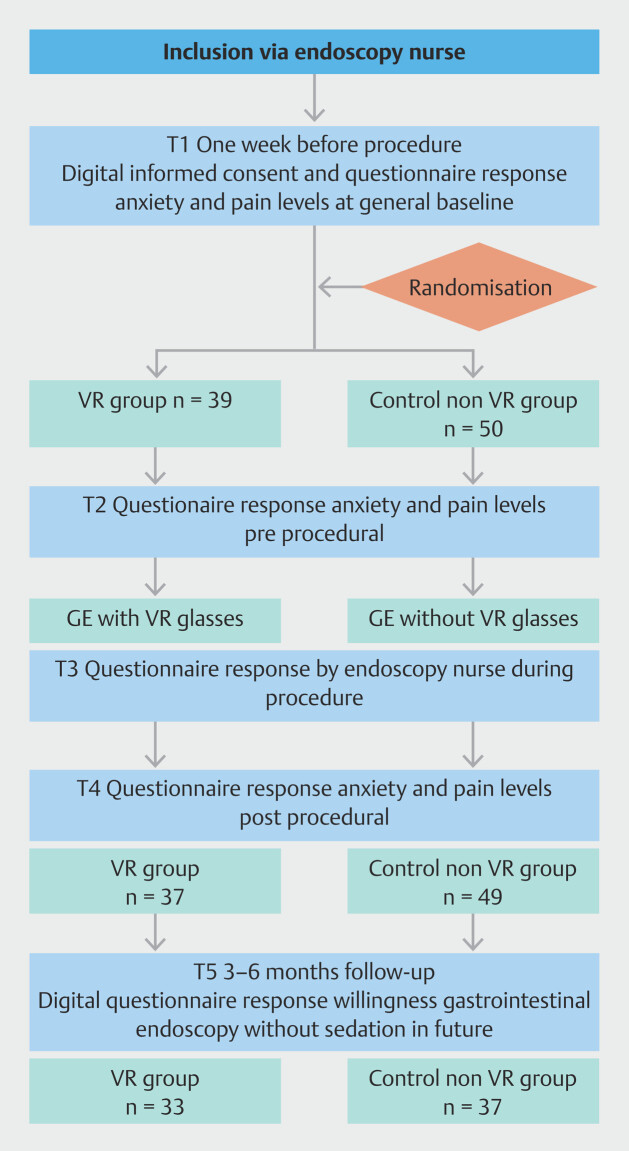
Study flowchart.

Between July 2021 and August 2023, 89 patients were enrolled in the study ( Fig. 1 ). Baseline characteristics of the study population were compared between groups to assess potential differences at study entry. Age, STAI baseline, and STAI trait baseline were analyzed as continuous variables and compared using univariate analysis of variance in that order: *F* (1, 87) = 0.92, *p* = 0.340, η²p =.01; *F* (1, 87) = 0.225, *p* =.636, η²p = 0.00; *F* (1, 87) = 0.29, *P* = 0.595, η²p =.00. Gender, prior experience with gastroscopy, and prior experience with VR were analyzed as categorical variables using the Chi-square tests in that order: χ²(1, *n* = 89) =.10, *P* = 0.922, OR = 0.96 95% [0.41–2.25]; χ²(1, *n* = 89) = 1.71, *P* = 0.191, OR 0.51, 95% CI 0.19–1.41; χ²(1, *n* = 89) = 0.66, *P* = 0.415, OR 1.56, 95% CI 0.52–4.83. Level of education and the RAND-36 subscales (namely physical functioning, social functioning, role limitations due to physical health, role limitations due to emotional problems, energy, emotional well-being, pain, general health and health change) were analyzed as an ordinal variable using Mann-Whitney U tests in that order: *U* = 879.5, *z* = -.86, *P* = 0.389, *r* = 0.09; *U* = 857, *z* = -0.99, *P* = 0.323, *r* = 0.11; *U* = 880, *z* = -0.80, *P* = 0.422, *r* = 0.085; *U* = 968.5, *z* = - 0.06, *P* = 0.953, *r* = 0.01; *U* = 922.5, *z* = -5.66, *P* = 0.572, *r* = 0.60; *U* = 974.5, *z* = -0.00, *P* = 0.997, *r* = 0.00; *U* = 973, *z* = -0.02, *P* = 0.987, *r* = 0.00; *U* = 952.5, *z* = -0.19, *P* = 0.851, *r* = 0.02; *U* = 1032.5, *z* =.48, *P* = 0.633, *r* = 0.05; *U* = 864, *z* = -0.98, *P* = 0.326, *r* = 0.10 ( Table 1 ).

**Table:** **Table 1** Baseline characteristics at time point T1.

**T1**	**Intervention VR** **N = 39**	**Control non-VR** **N = 50**	***P* value **	**Partial eta squared**
Age (years)	53.21 (16.36)	56.46 (15.50)	0.340	0.01
Gender (m:f)	23:16	30:20	0.922 ^‡^	0.96, 95% CI 0.41–2.25
Level of education (low: middle: high)	8:9:22	12:15:23	0.389 ^†^	0.09
STAI	36 (12.30)	37.28 (12.87)	0.636	0.00
STAI trait	38.46 (10.99)	37.20 (11.13)	0.595	0.00
RAND-36				
Physical functioning ^*^	90 [75–100]	90 [65–95]	0.323 ^†^	0.11
Social functioning ^*^	87.50 [62.50–100]	75 [62.50–100]	0.422 ^†^	0.09
Role limitations due to physical health ^*^	100 [0–100]	100 [0–100]	0.953 ^†^	0.01
Role limitations due to emotional problems ^*^	100 [100–100]	100 [66.67–100]	0.572 ^†^	0.60
Energy/fatigue ^*^	65 [40–75]	60 [43.75–80]	0.997 ^†^	0.00
Emotional well-being ^*^	80 [68–84]]	76 [63–88]	0.987 ^†^	0.00
Pain ^*^	77.55 [57.14–89.80]	67.35 [53.06–89.80]	0.851 ^†^	0.02
General health ^*^	60 [40–75]	60 [43.75–75]	0.633 ^†^	0.05
Health change ^*^	50 [25–50]	50 [25–50]	0.326 ^†^	0.10
Prior experience with gastroscopy (y:n)	7:32	15:35	0.191 ^‡^	0.51 95% [.19–1.41]
Prior experience with VR (y:n)	8:31	7:43	0.415 ^‡^	1.59 95% [.52–4.83]
^*^ Variables are denoted as median and lower and upper quartiles. ^†^ Mann Whitney U test. ^‡^ Chi square. CI, confidence interval; STAI, State-Trait Anxiety Inventory; VR, virtual reality.				

Of the patients randomized to undergo unsedated gastroscopy randomized for the study, 39 received the intervention with VR glasses. All patients in the intervention group used the VR glasses during the whole procedure. There were two endoscopists that performed all the procedures. After the procedure (T4) and 3-month follow up (T5), self-reported willingness to undergo unsedated vs sedated gastrointestinal endoscopy in the future was available respectively for N = 86 (97%) and N = 70 (79%) of patients.

### Primary outcome

**Fig. 2:**
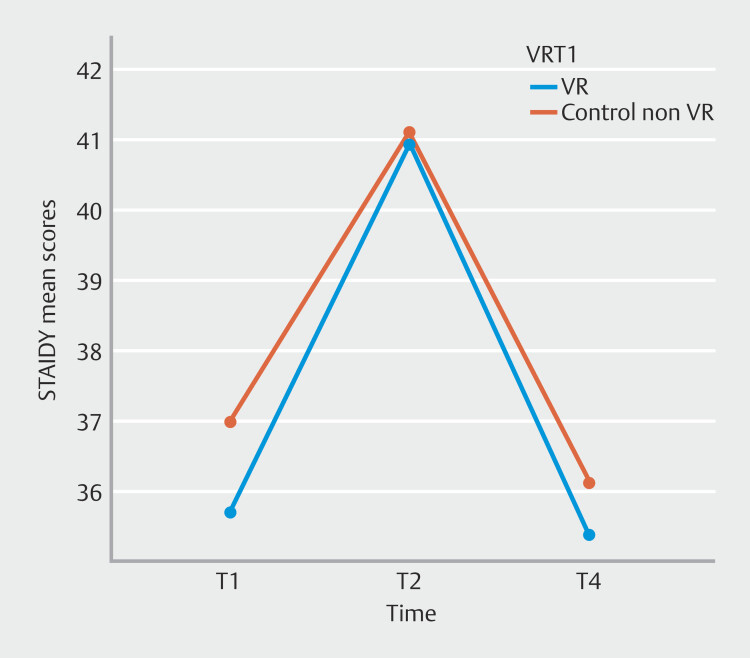
STAI DY mean scores. Anxiety levels before, during, and after gastroscopy.

The primary outcome was anxiety, assessed using the STAI questionnaire at three time points: T1 baseline, T2 immediately before gastroscopy, and T4 after gastroscopy. To evaluate changes in anxiety over time and between groups, a repeated measures ANOVA was performed. Baseline STAI scores at T1 did not differ significantly between groups (Table 1), and therefore, baseline scores were not included as a covariate in the analysis. Repeated measures ANOVA revealed no significant main effect of group (VR vs control non VR) on anxiety levels measured with the STAI anxiety questionnaire *F* (1, 84) = 0.11, *P* = 0.727, *r* = 0.00, which indicates that combined time scores for the STAI do not differ on average among the groups. There was a significant main effect of time on anxiety levels measured with the STAI anxiety questionnaire *F* (2, 83) = 16.23, *P* < 0.001, *r* = 0.28. Post hoc pairwise comparisons (Bonferroni--corrected) showed that anxiety levels were higher on T2 (immediately before gastroscopy) vs T1 and T4. T1 and T4 did not differ significantly. These results indicate that anxiety levels were higher immediately before baseline gastroscopy, and after gastroscopy, the levels dropped back to the level they were 1 week before the procedure for both groups ( Table 2 and Fig. 2 ). Most importantly, however, there was no significant interaction effect between group (VR vs control non VR) and time *F* (2, 83) = 0.123, *P* = 0.884, *r* = 0.00, demonstrating that the temporal pattern of anxiety was consistent across both groups and use of VR glasses did not significantly influence anxiety levels as measured with the STAI.

**Table:** **Table 2** Anxiety, pain, patient comfort, and willingness to return with VR and no sedation, displayed for the different time points T2, T3, T4, and T5.

**T2**	**Intervention VR** **N = 39**	**Control non-VR** **N = 50**	***P* value **	**Partial eta squared**
STAI-DY T2	40.97 (8.75)	41.04 (10.76)	0.975	0.00
NRS anxiety T2 ^*^	3 [2–7]	4 [1–6]	0.726 ^†^	0.04
NRS pain T2 ^*^	1 [0–4]	1 [0–3.25]	0.867 ^†^	0.02
T3				
Estimated comfort ^*^	7 [6–8.25]	7 [6–8]	0.599 ^†^	0.06
Medical findings (non-significant: significant: severe significant)	22:10:7	25:17:8	0.695 ^†^	0.04
Time in seconds of duration of passage	192.13 (57.75)	175.90 (60.83)	0.207	0.02
T4	N = 37	N = 49		
STAI-DY T4	35.38 (10.09)	36.12 (10.11)	0.736	0.00
NRS anxiety T4 ^*^	1 [0–4.5]	3 [1–4]	0.244 ^†^	0.13
NRS pain T4 ^*^	2 [1–4.5]	2 [0–4]	0.580 ^†^	0.06
Willingness to return without sedation T4 ^*^	8 [5–9]	8 [5–9]	0.940 ^†^	0.01
T5	N = 33	N = 37		
Willingness to return without sedation T5 ^*^	5 [2.5–8]	7 [2.5–9.5]	0.573†	0.07
^*^ Variables are denoted as median and lower and upper quartiles. ^†^ Mann Whitney U test. NRS, Numeric Rating Scale; STAI-DY, State-Trait Anxiety Inventory, Dutch version; VR, virtual reality.				

### Secondary outcomes

#### Procedure characteristics

All patients successfully completed gastrointestinal endoscopy. Procedural characteristics of the study population were compared between groups to assess potential differences at study entry. Medical findings (non-significant, significant, severe significant) were analyzed as an ordinal variable using Mann-Whitney U tests in that order: *U* = 1018, *z* = 0.39, *P* = 0.695, *r* = 0.04. Time in seconds of duration of passage was analyzed as a continuous variable and compared using univariate analysis of variance: *F* (1, 86) = 1.62, *P* = 0.207, η²p = 0.02 ( Table 2 ).

#### Patient anxiety and pain

Anxiety and pain scores are summarized in Table 2 . NRS for anxiety and pain were compared between the two groups (VR vs. No VR) at time points T2 immediately before gastroscopy and T4 after gastroscopy. Due to non-normal distributions, Mann-Whitney U tests were used for these comparisons. No significant differences were found between groups at T2 for anxiety NRS ( *U* = 933, *z* = -0.350, *P* = 0.726, *r* = 0.04) or pain NRS ( *U* = 955.5, *z* = -0.167, *P* = 0.867, *r* = 0.02). Similarly, at T4, there were no significant group differences for anxiety NRS ( *U* = 1038, *z* = 1.16, *P* = 0.244, *r* =0.13) or pain NRS ( *U* = 844, *z* = -.55, *P* = 0.580, *r* = 0.06).

#### Patient comfort and willingness to undergo unsedated vs sedated gastrointestinal endoscopy in the future

Scores for patient comfort and willingness to undergo unsedated vs sedated gastrointestinal endoscopy in the future are shown in Table 2. Patient comfort was measured at time point T3 during gastroscopy and willingness was measured at time points T4 after gastroscopy and T5 3-month follow up. All measures were compared between the two groups (VR vs. No VR). Due to non-normal distribution, Mann-Whitney U tests were used for these comparisons. No significant differences were found between groups for patient comfort ( *U* = 834.5, *z* = -0.53, *P* = 0.599, *r* = 0.06). Moreover, there were no significant group differences for willingness at T4 ( *U* = 898, *z* = -0.08, *P* = 0.940, *r* = 0.01) and T5 ( *U* = 658, *z* = 0.56, *P* = 0.573, *r* =.07) ( Table 2 ).

## Discussion

In this RCT, use of VR distraction did not result in a decrease in anxiety, pain, discomfort, or willingness to repeat gastroscopy without sedation. Moreover, mean anxiety levels increased 10 minutes before gastrointestinal endoscopy, and after the procedure, they dropped back to the previous level of 1 week before the procedure.

### Comparison with other studies

Because healthcare costs in the Netherlands are rising sharply, innovative methods to reduce costs are becoming more important. A recently published study on this topic by Boonreunya et al. (2022) showed that VR distraction did not reduce patient pain measures (VAS 0–10 scale), heart rate, or blood pressure during gastroscopy 14 . However, they did not include validated psychological tests in their study. Using these psychological tests, we attempted to demonstrate a difference in groups with or without VR distraction.

Numerous studies have shown that auditory and visual effects can reduce anxiety and increase satisfaction during EGD 9 . Moreover, Kim et al. (2023) revealed that VR exposure before endoscopic procedures may relieve patient anxiety levels measured with the STAI-state anxiety questionnaire prior to endoscopic procedures (patients with either EGD or colonoscopy) 13 . They reported that even 3 to 5 minutes of VR exposure prior to the endoscopic procedure reduced anxiety. Even though patients in our intervention group also wore VR glasses before gastroscopy for 10 minutes to get accustomed to them, these results are contrary to our findings. Namely, in our study, mean anxiety levels increased 10 minutes before the procedure in both groups.

VR distraction has proven to be effective in reducing procedural pain in several medical procedures, such as wound care, rehabilitative physical therapy, chemotherapy, surgery, and dental treatment 23 . Possibly, distraction techniques have a limited and finite ability to lessen anxiety, pain, and comfort during gastroscopy. It is conceivable that the throat gag reflex during gastroscopy is such a strong autonomous response that VR distraction alone may not be effective. Follow-up research may focus on use of VR distraction in combination with selective anesthesia on the throat. This combination may provide a cost-saving alternative to undergoing gastroscopy with sedation. Moreover, although the current findings do not support general use of VR distraction during gastroscopy, it remains possible that specific subgroups of patients may derive clinical benefit. Characteristics such as age, personal traits, anxiety, or previous endoscopy experiences could potentially influence individual responsiveness to VR distraction. Future studies, therefore, could focus on identifying these subgroups, enabling more effective application of this intervention. In the meantime, if VR distraction is offered despite the lack of robust evidence, it is important that patients are adequately informed about the current group-level findings so they can make well-informed decisions about use of VR distraction.

### Limitations

Some limitations have to be acknowledged. First, selection bias cannot be ruled out because a proportion of patients chose sedation after receiving information about the procedure and this study. This self-selection may have influenced composition of the study groups and potentially affected generalizability of the findings. Second, blinding of patients was not possible due to the nature of the intervention, which may have introduced bias related to patient expectations. Although blinding of endoscopy nurse could have been implemented by having all patients wear VR glasses – with only some receiving visual and auditory stimuli – this approach was not applied in the current study. Future research might consider such strategies to minimize potential observer bias. A further limitation is that we did not perform cost calculations. In particular, indirect economic consequences, such as potential loss of productivity after sedation compared with immediate resumption of activities after unsedated gastroscopy, were not calculated in our analysis. Future research could address these aspects to provide a more complete assessment.

### Strengths

Strengths of this study include the randomized design and measures that were based on predecessor studies of various procedures, which increases comparability. In addition, we used well-validated psychological questionnaires to complement one previous study on VR distraction during gastrointestinal endoscopy.

## Conclusions

Our results show that VR distraction did not reduce patient anxiety, pain, or discomfort before, during, or after gastrointestinal endoscopy. Moreover, willingness to undergo the same procedure without sedation was the same for the VR and control groups. Thus, our findings do not support a general benefit of VR distraction in reducing discomfort during unsedated gastroscopy. Further studies are needed to investigate whether there is a clinical benefit of VR distraction during gastroscopy. More specifically, future studies could focus on exploring whether selected groups of patients may benefit from VR distraction. Also, VR distraction can be studied in combination with selective anesthesia on the throat to explore whether it may provide a cost-saving alternative to undergoing gastroscopy with sedation.

## Ethical approval

The research protocol was approved by the Local Review Commission (LTC) of Canisius Wilhelmina hospital (047–2021). All patients provided digitally informed consent before enrolment.

## Affirmations

The lead author (the manuscript’s guarantor) affirms that the manuscript is an honest, accurate, and transparent account of the study being reported; that no important aspects of the study have been omitted; and that any discrepancies from the study as planned have been explained.
